# Suicide Risk Analysis and Psycho-Emotional Risk Factors Using an Artificial Neural Network System

**DOI:** 10.3390/healthcare11162337

**Published:** 2023-08-18

**Authors:** Francisco Manuel Morales-Rodríguez, Juan Pedro Martínez-Ramón, José Miguel Giménez-Lozano, Ana María Morales Rodríguez

**Affiliations:** 1 Department of Educational and Developmental Psychology, Faculty of Psychology, University of Granada, 18011 Granada, Spain; josemiguelgimenez92@gmail.com; 2 Department of Evolutionary and Educational Psychology, Faculty of Psychology and Speech Therapy, Campus Regional Excellence Mare Nostrum, University of Murcia, 30100 Murcia, Spain; juanpedromartinezramon@um.es; 3 Department of Journalism, Faculty of Communication Sciences, University of Málaga, 29010 Malaga, Spain; amoralesr@uma.es

**Keywords:** suicide, suicide risk, youth, artificial neural network, artificial intelligence, protective and risk factors

## Abstract

Suicidal behavior among young people has become an increasingly relevant topic after the COVID-19 pandemic and constitutes a public health problem. This study aimed to examine the variables associated with suicide risk and determine their predictive capacity. The specific objectives were: (1) to analyze the relationship between suicide risk and model variables and (2) to design an artificial neural network (ANN) with predictive capacity for suicide risk. The sample comprised 337 youths aged 18–33 years. An ex post facto design was used. The results showed that emotional attention, followed by problem solving and perfectionism, were variables that contributed the most to the ANN’s predictive capacity. The ANN achieved a hit rate of 85.7%, which is much higher than chance, and with only 14.3% of incorrect cases. This study extracted relevant information on suicide risk and the related risk and protective factors via artificial intelligence. These data will be useful for diagnosis as well as for psycho-educational guidance and prevention. This study was one of the first to apply this innovative methodology based on an ANN design to study these variables.

## 1. Introduction

Suicide is a difficult topic to understand in the current society, even for psychologists, scientists, and researchers who study and deal with it on a daily basis. “How can someone take their own life leaving everything behind? How can they lose so much hope? How can they stop fighting? There is always a way out..., there has to be..., right?” Many questions, whys, and wherefores exist and require a deeper analysis. Abundant factors, which can be infinite, may play an influencing role [1]. Additional questions can be added if the person who has exercised their option to leave freely and deliberately is an adolescent, given the emptiness and great pain it causes their families.

Durkheim (1928), one of the first to scientifically define suicide, defined it as “any case of death resulting, directly or indirectly, from a positive or negative act, carried out by the victim himself, knowing that it would produce this result” [2]. When dealing with suicide, certain theoretical obstacles need to be addressed before an adequate analysis can be made. There is consensus when it comes to understanding the act of suicide as the behavior taken by a person with the intention of attempting to take their own life as a response to multiple factors [3]. Villalobos (2009) states that for an act of suicide to occur, three types of determinants must be met: death as a result of harm, which has been produced by the victim themselves and intentionally inflicted [4]. Conversely, there is still confusion regarding some terminologies associated with suicide.

Once suicide has been defined, suicidal ideation arises. Llosa-Martinez and Canetti-Wasser (2019) defined suicidal ideation as the thought of the will to take one’s own life, the intrapsychic element that leads a person to commit such an act [5]. Furthermore, O’Carroll et al. (1996) stated that it could only be detected verbally through self-report [6]. Researchers warn that suicidal ideation is a highly significant predictor prior to the behavioral act of suicide [7]. Hence, they stress the importance of performing quality follow-ups that detect such thoughts in advance.

Conversely, attempted suicide would be any failed act intended to take one’s own life by intentionally inflicting harm [8]. This type of self-injurious action would involve suicidal ideations and plans to commit suicide; however, for one reason or another, it is not achieved [9]. In addition, suicidal risk is the sum of harmful factors (social, family, psychological, and behavioral) that a person suffers and also increases the likelihood of suicide attempts and subsequently may lead to suicide [10].

In this work, the modeling approach that was followed is the one proposed in the theoretical framework indicated by Gabilondo [11]. In coherence with the SESPAS Report (Spanish Society of Public Health and Health Administration), the need arises to follow a multidisciplinary model for suicide prevention in Spain, promoted by the World Health Organization (WHO), which emphasizes the multifactorial nature of suicide and in which different agents such as educational agents must be involved rather than focus only on a healthcare—health model. In this sense, cultural factors are considered in this model as critical risk factors. Specifically, the university setting is a relevant transversal educational agent for suicide prevention, and more evaluations must be conducted to raise awareness and train more effectively for suicide prevention.

It is necessary to differentiate between risk factors related to suicidal ideation and those related to completed suicide. Regarding suicidal ideation, the psycho-emotional dimension that includes variables such as hopelessness or grief in interaction with factors of other nature may be related to suicidal ideation [12]. Similarly, the negative aspects of the emotional dimension, specifically restlessness and mood disorders such as anxiety and depression, are often symptoms involved before suicidal ideation [13,14], and these specific symptoms can be affected by social isolation or low-quality social interactions [15,16] and by using a coping strategy that is more or less productive depending on the situation [17]. In this regard, a recent study [18] found that the perceived social support strategy [18] is one of the protective factors of suicidal ideation. Romero-Acosta [19] found that the experience of victimizing events in the family environment is another risk factor for suicidal ideation in children and adolescents. Miranda-Mendizabal et al. [20] found that specific risk factors for suicide attempts for males were the following: (a) hopelessness, (b) behavioral problems, (c) access to media, (d) suicidal behavior of a friend, and (e) parental divorce or separation. In addition, Miranda-Mendizabal et al. [20] found that specific risk factors for suicide attempts for women were the following: (a) post-traumatic stress disorder, (b) eating disorders, (c) having been a victim of dating violence, (d) interpersonal problems, (e) previous abortion, (f) depressive symptomatology, and (g) bipolar disorder.

Regarding the risk of consummated suicide in the future, one of the relevant predictors noted is suicide attempt [21,22,23] and anxiety disorder and depression [14]. Specifically, in a recent study by Aiartzaguena and Morentin [21] conducted in Spain on the risk factors for completed suicide in a sample aged between 14 and 55 years, the rate was three times higher in men, increasing with age with respect to demographic factors. Similarly, Aiartzaguena and Morentin [21] found that the risk factors for completed suicide in young people were as follows: clinical risk factors such as mental and mood disorders such as depression (72%); disorders due to substance use (20.5%) and psychotic disorders (14%); and other factors such as suicide attempt (20.5%), physical illness (23%), and sociofamilial risk factors (9%). The prevalence of alcohol and drug use and substance abuse disorder was higher in women than in men [21]. Miranda-Mendizabal et al. [20] pointed out that further research on risk factors for suicide death for women is needed. This study also found that for men, the specific risk factors for death by suicide were access to media; drug abuse; and so-called externalizing disorders such as aggression, delinquency, and antisocial behaviors [21].

When discussing predictors of suicide risk, whether it is the risk for suicidal ideation, attempted suicide, or completed suicide, it is necessary to address other relevant psychological factors, such as the role of drugs [14,24] and smoking [25]. In this regard, in the meta-analysis conducted by Poorolajal et al. [24], in which the association between substance use disorder and suicidal risk was examined, statistically significant associations were found between substance use disorder and suicidal ideation, suicide attempt, and suicide death. In addition, further research is needed to investigate the relationships between suicidal risk and specific types of illicit drugs. Echeverria et al. [25] also recently conducted a meta-analysis with the aim of evaluating the relationships between smoking and suicidal behavior, finding associations between exposure to tobacco (whether current or past smokers) and increased risk for suicidal behavior, whether planning or attempting suicide, suicide intent, or death by suicide. In this sense, these authors suggest that intervention for and assessment of smoking would also be necessary as a preventive strategy for suicidal behavior. In both cases, in terms of ideation and completed suicide, the importance of the care received from an adequate health policy must be pointed out, as well as how cultural variables must be considered [11,26], as a certain factor can have more or less weight in a given culture or context. Similarly, in a meta-analysis [20] of a sample of adolescents and young adults aged 12–26 years (a normative nonclinical population), both males and females, substance abuse disorder, mental disorder, and exposure to interpersonal violence were found to be common risk factors for suicidal behaviors.

Detecting the multiple factors surrounding suicide is extremely important. According to the Spanish National Institute of Statistics [27], suicide was the primary cause of external deaths (which included accidents, falls, assaults, and homicides) in 2021, with 4003 deaths, of which 2982 were men. Statistics suggested that suicide in early life, such as in adolescence, was the leading cause of unnatural deaths in Spain between those aged 15–29 years [28]. In 2021, the ANAR Foundation [29] conducted a study via a telephone call with 748 minors who, at the time of the call, were attempting to take their own lives. Furthermore, they exposed that since 2012, when they started the campaign to now, a significantly exponential increase in cases of suicidal behavior was observed (1921.3%). The post-COVID-19 period that lasted from 2020 to 2022 stood out the most. These data contrasted with those collected by the FAD Foundation and the Queen Sofia Center [30], which found that more than half of adolescents or young adults (56%) had verbalized having a delicate mental health condition, and practically half had not sought professional help (49%). This report also indicated that women reported more psychosocial problems than men, with a difference of almost 20%.

Therefore, being female may be a risk factor that would increase the probability of committing a suicidal act. However, Gonzalez-Sancho and Picado-Cortés reported that the various risk factors involved among adolescents could be classified into two large blocks: (a) psychosocial risk factors and (b) risk factors due to biological or psychiatric conditions [31]. Within the first block, the authors created three subgroups: (a) in relation to their peers, (b) family dynamics, and (c) media. Other authors further added to these types, such as those related to negative experiences (bullying) [32] and even spending more than three hours a day connected to the Internet [33].

Studies reported that stigmas related to the LGTBI community were prevalent risk factors of psychosocial nature owing to the dichotomy of a human being a man and a woman, and predominantly heterosexual. Furthermore, acts of rejection and violence against those who do not follow these guidelines may cause anxiety, depression, or low self-esteem among them [34]. Studies reported that rejection by their peers and the rest of society was an important risk factor among LGTBI individuals, given the stage of maturational and vital development that causes the creation of networks beyond those created within the family environment [35]. Some related factors include null or scarce communication with others, school problems, unwanted pregnancies, lack of emotional resources available to face rejections, and absence of stable affective relationships [36]. The effect of bullying on suicidal acts, as well as school bullying in person and through social networks (cyberbullying), are important [37]. The ANAR Foundation reported that 70% of the adolescents evaluated and who presented suicidal ideation at some point declared having suffered mistreatment at school and/or high school [29]. Bailin et al. stated that cyberbullying could generate cases of depression and functional alterations in school, work, personal hygiene, and/or self-efficacy [38]. The weight that the media has in society is noteworthy. Hence, studies have evaluated the influence media can have on the contagion effects or “Werther effect”, where the way of reporting the news could influence suicidal behaviors among other adolescents [39].

Other factors would include those of a psychological or biological nature. Studies on biological factors are scarce, with limited comprehensive results to reach a clear conclusion; however, a statistically significant correlation was observed that conditions in the amygdala or prefrontal cortex, even certain genetic anomalies, were correlated with increased suicide risk [40]. Psychological factors have been most researched. Many studies have related suicide attempts and the act itself to psychiatric illnesses [41,42]. Major depressive disorders in adolescents were a direct cause of suicide [43]. Furthermore, the probability increased with other related factors, such as an anxiety crisis [44], panic attacks [45], or post-traumatic stress attacks [46]. Some studies observed alterations in emotional processing or even decision making (neural ability within the executive functions) [47] and coping strategies [48].

The modeling approach used, as compared with more commonly analyzed models in psychology (e.g., multiple regression and ordinal logistic regression), has advantages since it allows the design of artificial neural networks to analyze the relationships between psycho-emotional constructs and suicidal risk. This aims to clarify, through the proposed model, the previous results obtained from other types of analysis that are still not entirely conclusive. This could help design more effective cross-cutting programs, especially in university settings, for the prevention of suicide risk factors and the promotion of protective factors with the assistance of an “intelligent tutor” from the university teaching environment [49,50]. Additionally, it could aid decision-makers in health policies and designing programs for prevention and psycho-educational intervention, leveraging the improved predictive capacity and other advantages of artificial intelligence in this field [51,52]. For instance, it could enable even more personalized and effective learning experiences [53], which is crucial when dealing with aspects related to health education.

Despite the above-mentioned studies, further studies are required to evaluate young ages and confirm inconclusive or contradictory results via an innovative methodology based on an analysis of artificial neural networks to examine whether certain previous results related to these variables, such as the emotional dimension either in its negative or positive pole, were confirmed. This study aimed to examine the psycho-emotional variables associated with suicidal risk and determine their predictive capacity. The specific objectives were: (1) to analyze the relationship between suicidal risk and variables of the model and (2) to design an artificial neural network with predictive capacity of suicidal risk.

## 2. Method

### 2.1. Design and Procedure

This was a quantitative, cross-sectional, ex post facto design study. A battery of questionnaires was administered to university students from different degrees after the study procedure and purpose were described, and informed consent was obtained. The language used in the questionnaires was Spanish. The sampling method was convenience or accidental sampling [54]. Depending on availability or scope in the workplace, students from the Faculties of Psychology and Educational Sciences were invited. We ensured that students did not feel pressured to participate or demonstrate acquiescence bias to please teachers since the answers were anonymous. In most cases, they did not know the teacher who invited them to participate; they could leave at any time and stop answering. Validated instruments with appropriate questions were also applied in that regard. It took approximately 30–45 min to complete the battery. Participants were allowed to withdraw at any time without adverse consequences. The research was conducted following the guidelines of the Declaration of Helsinki for research with humans and was voluntary, confidential, and anonymous. Likewise, this study was approved by the university’s ethics committee (3520/CEIH/2023).

### 2.2. Participants

The sample consisted of 337 university students aged 18–33 years (*M* = 20.22 years, *SD* = 2.542). Table 1 shows the main indices of dispersion and the central tendency of the quantitative non-sociodemographic variables. Of the participants, 14.2% were male, and 22.8% were enrolled in their first year. Regarding the degree, 11.6% (39), 45.7% (154), and 5.9% (20) were in psychology, primary education, and pedagogy degrees, respectively. Furthermore, 36.8% (124) constituted missing data from degrees belonging to education sciences and psychology. Of the participants, 11% (37) claimed they had a partner, and 0.3% were married (1). Furthermore, 26.1% had sisters or brothers. The sample was selected via incidental sampling. Young people with disabilities or severe behavioral disorders were excluded if their situation did not allow them to participate.

### 2.3. Instruments

#### 2.3.1. Depression, Anxiety, and Stress Scale—21 Items (DASS-21) [55,56]

This scale was composed of three dimensions that measured depression, anxiety, and stress on a 4-point Likert-type scale that ranged from 0 (did not apply) to 3 (applied most of the time in the past week). Total scores ranged from 0 to 21 points. The higher the score, the greater the symptomatology. In this study, the scores of the depression dimension (sum of items 3, 5, 10, 13, 16, 17, and 21) were analyzed, and a Cronbach’s alpha of 0.907 was obtained. An example of an item was, “I could not feel any positive feelings” (item 3). The Spanish version of the DASS-21 was administered [57].

#### 2.3.2. Plutchik’s Suicide Risk Scale (PSRS) [58]

This scale comprised 15 items and two response options: yes or no. Positive and negative responses were scored one and zero points, respectively. The higher the score, the higher the risk of suicide. A score of ≥6 implied a positive assessment of suicidal risk. In this study, Cronbach’s alpha was 0.868. An example of an item was, “Do you regularly take any medication, such as aspirin or sleeping pills?” (item 1). The Spanish version of the PSRS was administered [59].

#### 2.3.3. Perceived Stress Scale (PSS) [60]

This scale comprised 14 items that measured the participants’ perception of stress in the last month. Responses were rated on a 5-point Likert scale that ranged from 0 (never) to 4 (very frequently). Scores of all the items were summed. The higher the score, the higher the perceived stress. In this study, Cronbach’s alpha was 0.788. An example of an item was, “In the last month, how often have you been affected by something that has happened unexpectedly?” (item 1). The Spanish version of the PSS was administered [61,62].

#### 2.3.4. Generalized Self-Efficacy Scale (GSES) [63,64]

This scale measured self-efficacy through 10 items on a 4-point Likert scale that ranged from 1 (never) to 4 (always). An example of an item was, “I can find a way to get what I want, even if someone opposes me” (item 1). The higher the score, the higher the self-efficacy. The total score was calculated. In this study, Cronbach’s alpha was 0.884. The Spanish version of the TMMS was administered [63,64].

#### 2.3.5. Trait Meta-Mood Scale (TMMS) [65]

This scale measured emotional intelligence through three dimensions: emotional attention (α = 0.903), emotional clarification (α = 0.886), and emotion repair (α = 0.878). In total, 24 items were rated on a 5-point Likert scale that ranged from 1 (do not agree at all) to 5 (strongly agree). An example of an item was, “I pay a lot of attention to feelings” (item 1). The Spanish version of the TMMS was administered [66].

#### 2.3.6. State-Trait Anxiety Inventory (STAI) [67]

This inventory was divided into two scales: state anxiety (α = 0.934) and trait anxiety (α = 0.759). An example of an item was, “I feel calm” (item 1). State anxiety consisted of the first 20 items, and the remaining 20 were on trait anxiety, which totaled 40 items. Responses were rated on a 4-point Likert scale that ranged from 0 (not at all and almost never) to 3 (very much and almost always) for state and trait anxiety, respectively. The Spanish version of the STAI was administered [68,69].

#### 2.3.7. Coping Strategies Inventory [70,71]

The coping scale comprised eight dimensions, each of which accounted for a specific coping strategy: problem solving (REP; α = 0.882), self-criticism (AUC; α = 0.910), emotional expression (EEM; α = 0.863), cognitive restructuring (REC; α = 0.864), desiderative thinking (PSD; α = 0.867), social support (APS; α = 0.8589), problem avoidance (EVP; α = 0.772), and social withdrawal (RES; α = 0.790). It consisted of 40 items plus one extra item rated on a 5-point Likert scale that ranged from 0 (not at all) to 4 (totally). An example of an item was, “I struggled to solve the problem” (item 1). The Spanish version of the TMMS was administered [69,70].

#### 2.3.8. Multidimensional Perfectionism Scale [72]

This scale measured the level of perfectionism via 35 items on a scale from 1 to 5. An example of an item was, “If someone does a task at school/work better than me, I feel as if I have totally failed at that task” (item 13). In this study, Cronbach’s alpha was 0.940. The Spanish version was administered [73].

#### 2.3.9. Escala de Personas Altamente Sensibles—Versión Española (Spanish Version of Highly Sensitive Person Scale; HSPS-S) [74]

This scale measured high sensitivity—closely related to sensation processing—in people via 27 items. Responses were rated on seven options, where 1 meant “strongly disagree” and 7 referred to “strongly agree”. In this study, Cronbach’s alpha was 0.923. An example of an item was, “Are you easily overwhelmed by strong sensory stimuli?” (item 1).

### 2.4. Data Analysis

A descriptive analysis was performed for percentages, frequencies, main dispersion indexes, and central tendency. An inferential analysis was also performed. Specifically, Pearson’s correlation was applied to determine the degree of the association between two variables of a continuous or scaled nature and Student’s *t*-test, together with Levene’s F-test, for differences between means of the independent samples. Suicide risk was considered as a grouping variable. Finally, a backpropagation algorithm artificial neural network (ANN) was trained to study the contribution of the independent variables of the input layer to the power of the ANN to predict the location of the cases in one of the two categories of the dichotomous variable (suicidal risk understood as present (1) or absent (0)). A three-layer model was selected for its usefulness in generating complex relationships between nodes and synaptic weights that ultimately generate adequate predictive capacity. Nodes (neurons) are processing units that form the layers and process the information in the network. Each node has a specific function and performs mathematical operations on the received data. Synaptic weights are adjustable parameters associated with the connections between nodes and determine the strength and direction of the influence that one neuron has on another. These weights are adjusted during training to improve performance and allow the network to learn and adapt from the training data [75]. In this research, a multilayer ANN Perceptron artificial neural network was designed based on three layers: input capable, hidden layer, and output capable. In an ANN, the layers represent hierarchical levels of processing that allow the transformation and representation of complex data. The input layer is the first stage, which is responsible for receiving the initial signals and representing the characteristics of the data set. The hidden layers, which are in between, perform internal computations through weighted connections between neurons, allowing learning and discovery of relevant patterns in the data. Finally, the output layer produces the final answers or predictions based on the information processed by the previous layers [76,77]. Previously, a random seed with fixed value = 96,485 (integer part of Plank’s constant) was inserted. The input layer was composed of the independent variables of the network. The output layer consisted of a dichotomized continuous variable. The analysis of the cases was distributed between a training phase (in which a learning algorithm is used), a testing phase (in which errors are corrected), and a holdout phase (which is used to fully evaluate the final performance of the model once it has been trained and tuned with data that have not been used in any of the previous phases, thus providing a more realistic assessment of how the model generalizes to new and unseen data) [75,76,77]. During network programming, an approximate case distribution of 7, 2, and 1 out of 10 was proposed for the training, testing, and holdout phases, respectively. In total, 63.3% of the cases were used in the training phase by the network. Furthermore, 22.4% were assigned to the testing phase. Finally, 14.3% were used in the reserve phase. Within the input layer, the gender factor was used, and the following covariates were introduced: age, depression, perceived stress, self-efficacy, emotional attention, emotional clarity, emotional clarity, emotional clarity, emotion repair, state anxiety, trait anxiety, problem solving, self-criticism, emotional expression, desiderative thinking, social support, cognitive restructuring, problem avoidance, social withdrawal, perfectionism, and high sensitivity. In total, 21 units made up the input layer. The input and hidden layers were also made up of a bias unit or node. The scaling method for the covariates was normalized. The number of hidden layers was one—plus the bias node—and the number of units in the hidden layer was also one, with the activation function being the hyperbolic tangent. As for the output layer, the number of units was 2, the activation function was softmax, and the error function was cross-entropy. Eventually, to assess the ANN, the Receiver Operating Characteristic or ROC curve was used. This is a graphical representation for evaluating the performance of a binary neural network classifier by comparing sensitivity (true positive rate) with specificity (true negative rate) at different decision thresholds. The diagonal connecting the points (0,0) and (1,1) on the graph represents the expected performance of a random classifier. Any model with a curve below this diagonal is worse than a random model. The point closest to the upper left corner of the graph represents the optimal point for the classifier, where maximum sensitivity and specificity are achieved simultaneously. However, the area under the ROC curve (AUC) is used to quantify the overall performance of the classifier. Being a value between 0 and 1, the higher the AUC, the better the performance of the model. An AUC of 1 indicates a perfect model, while an AUC of 0.5 suggests that the model has no discriminative ability. At this point, the model offers the best balance between the detection and discrimination of positive and negative cases [78]. The software used was SPSS version 28.0.

## 3. Results

### 3.1. Analysis of the Relationship between Suicidal Risk and the Variables under Study

Table 1 shows the main statistics for the non-sociodemographic quantitative variables.

Regarding the dependent variable of the artificial neural network model “suicidal risk”, 37.7% (*n* = 127) were in the “yes” category, and 62.3% (*n* = 210) were in the “no” category, with no missing cases.

Regarding the relationships between the variables, Table 2 shows the Pearson correlations of the 95% confidence intervals related to suicide risk.

Table 3 presents the descriptive analysis of the scores obtained in the different variables according to whether they presented suicidal risk or not.

A *t*-test was performed for the equality of independent samples’ means as a function of the presence or absence of suicidal risk. Significant differences were found between the variables, as shown in Table 4. Cohen’s d-point estimate used the pooled standard deviation.

As shown in Table 3 and Table 4, when analyzing the profile of those who presented a suicidal risk, statistically higher levels of perceived stress, romantic solitude, emotional care, perfectionism, and high sensitivity were found. Lower levels of anxiety state, trait anxiety, and self-criticism were also found in people with no suicidal risk.

### 3.2. Design of an Artificial Neural Network with Predictive Capacity for Suicide Risk

**Figure 1:**
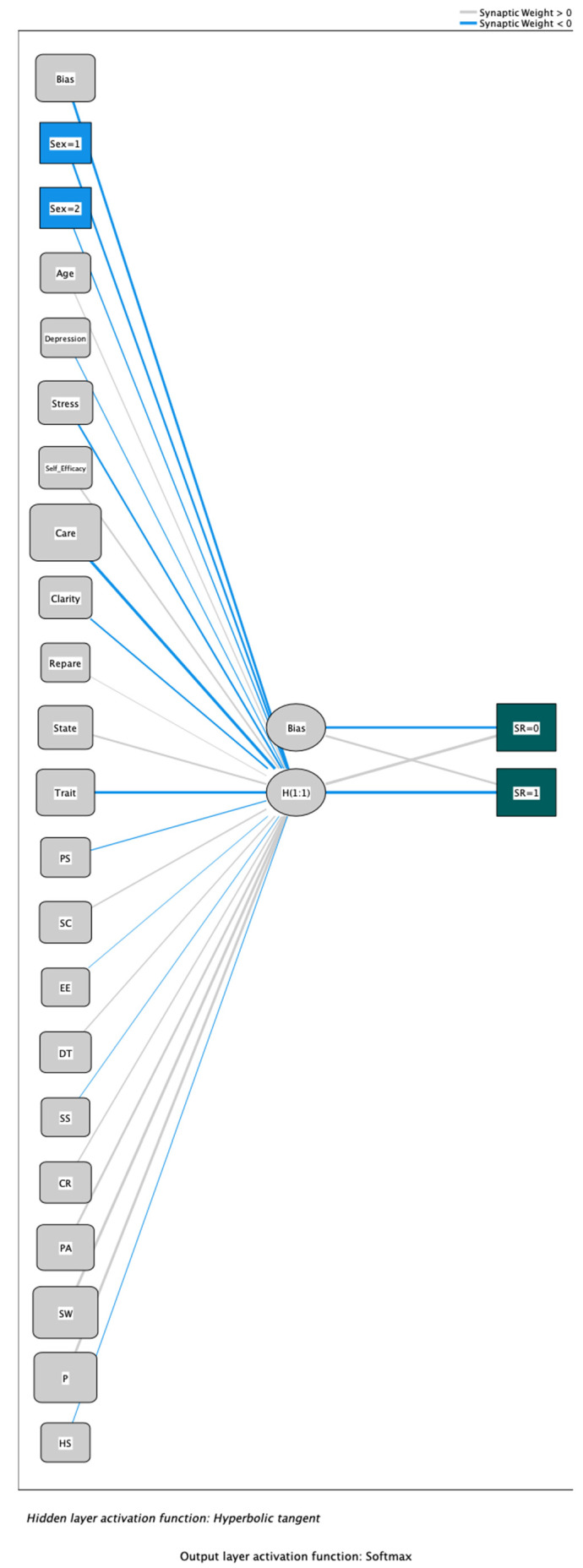
Representation of the ANN (DV: Suicidal risk). Note: Sex = 1: Male; Sex = 2: Female; Care: Emotional care; Clarity: Emotional clarity; Repair: Emotional repair; State: Anxiety state; Trait: Anxiety trait; PS: Problem solving; SC: Self-criticism; EE: Emotional expression; DT: Desiderative thinking; SS: Social support; CR: Cognitive restructuring; PA: Problem avoidance; SW: Social withdrawal; P: Perfectionism; HS: High Sensitivity. SR = 0: absence of suicidal risk; SR = 1: presence of suicidal risk. Source: Own elaboration.

Figure 1 shows the synaptic weighting and relationships between the nodes of the different layers graphically. The central nodes have been considered components of one of the three layers of the model, called the hidden layer.

Regarding the parameter estimates, in the training phase, the cross-entropy error was 14.410, the percentage of incorrect predictions was 14.5%, and the stopping rule used was that of a consecutive step with no decrease in error. Regarding the testing phase, the cross-entropy error was 2.764, and the percentage of incorrect predictions was 4.5%. Third, in the reserve phase, the percentage of incorrect predictions was 14.3%, and the dichotomized dependent variable was suicidal risk. Conversely, Table 5 shows the parameter estimates with the values of the synaptic weights.

Table 6 shows the importance of the independent variables or their contribution to the predictive ability of the ANN. Of the 20 variables analyzed in the input layer as independent variables of the model, the ten with the highest predictive capacity were the following, from highest to lowest contribution: emotional care, social withdrawal, perfectionism, trait anxiety, problem avoidance, anxiety state, total perceived stress, self-efficacy, emotional clarity, and self-criticism. Table 7 shows the classification of cases by phase.

**Figure 2:**
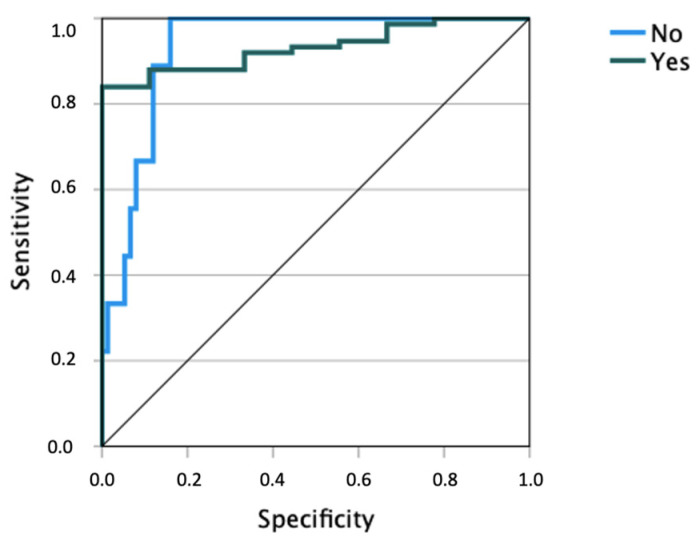
Sensitivity versus specificity curve of the ANN (dependent variable: suicide risk). Source: Own elaboration.

The ROC curve delineates an area beneath the curve where sensitivity and specificity are optimized (Figure 2). On another note, an AUC equal to 0.5 indicates a performance equivalent to chance, while an AUC close to 1 indicates a very effective model. In this research, AUC = 0.932 was achieved, which means the model has a strong ability to differentiate between the positive and negative classes, making it reliable for the task it was designed for. Such a high AUC is indicative of a model that has been well-trained and is likely to make accurate predictions on unseen data (see Figure 2)

## 4. Discussion

This study aimed to examine the relationship between suicidal risk and other protective and risk factors by designing an artificial neural network with predictive capacity for suicidal risk. Emotional attention had the highest predictive capacity in the ANN, followed by the problem solving coping strategy, level of perfectionism, and trait anxiety. Hence, the findings suggest that key independent variables influencing suicidal risk include emotional intelligence, coping mechanisms, anxiety, and the degree of perfectionism.

Specifically, the emotional care dimension related to the ability to express and experience feelings appropriately shows the highest predictive ability in the artificial neural network-based model. This result is congruent with a previous study [79] conducted in another context in which a dual model based on RNN neural networks and the analysis of open vocabulary, such as emotional lexical expressions and other characteristics that are a part of the emotional dimension, such as emotional stability. In that study [79], a greater use of the emotional dimension in its negative aspect, such as a more depressive and anxious language, was found to correlate with a higher level of suicidal risk. Similarly, this study found that users with greater anger as an emotion scored higher on the suicide risk variable. Taliaferro et al.’s [80] study involving 70,022 students found that hopelessness was one of the risk factors for both suicidal ideation and behavior.

Regarding coping strategies, the use of productive coping strategies, such as problem-solving skills and cooperative skills, as in our study, are considered protective factors for suicidal risk [81]. Similarly, among the functional strategies, in Mirkovic et al.’s [81] recent study, the following are also noted: “working hard and achieving”, “physical recreation”, and “seeking relaxing diversions”. Other research [82] highlights that communication problems and difficulties in problem solving with the presence of family conflicts would be risk factors for suicidal behavior. Similarly, Taliaferro et al. [80] found that parent connectedness is one of the protective factors. However, the scientific literature [17] has established that the use of strategies considered a priori as dysfunctional or unproductive, such as social withdrawal and problem avoidance, are generally associated with greater internalized symptomatology, such as anxiety and depression, which is one of the risk factors for suicidal ideation as indicated by Mata et al. [83].

Regarding the emotional dimension in its negative aspect of anxiety, the results of our study are consistent with Matero et al. [79], in which statistically significant associations were found between levels of anxiety and depression with a higher level of suicidal risk. Similarly, a meta-analysis and systematic review of longitudinal studies conducted by Gili et al. [14] found that an anxiety disorder is one of the important risk factors for suicide; therefore, its prevention in combination with other factors is critical for, for example, suicidal ideation to progress to suicide attempt in young people.

Regarding the perfectionism variable, another recent research [83], as in the present study, found that the level of perfectionism is another relevant factor for suicide risk. Specifically, Mata et al. [83] evaluated suicidal ideation, level of perfectionism, depressive symptomatology, and negative life events in 224 Portuguese young people aged 18–25 years; they found that the level of perfectionism was one of the risk factors that mediated the relationship between negative life events and suicidal ideation; they pointed out the importance of assessing levels of perfectionism for the prevention of suicidal behavior. In addition, Roxborough et al. [84] found that so-called maladaptive perfectionism was associated with the level of suicidal ideation and that negative life events constitute another important risk factor for suicidal behavior. Leal [85] also found statistically significant correlations between the level of maladaptive perfectionism and the tendency for suicidal ideation. Chemisquy [86] also highlights relationships between so-called unhealthy or maladaptive perfectionism and the variable suicidal ideation.

Studies that address the potential uses of ANN, such as its utility in the design of prevention and monitoring programs, are emerging. The ANN achieved a hit rate of 85.7%, much higher than chance, and with only 14.3% of incorrect cases. These results were congruent with those of other previous studies where the emotional dimension was relevant [43,44,46,47]. However, this study confirmed and clarified previous results based on the current possibilities for psychology offered by artificial intelligence. The variable sex was an important independent variable of ANN. Some previous studies found higher levels of anxiety and predisposition to suicidal risk in women than in men [87,88]. Therefore, this variable should be considered in the design of future programs for the prevention of suicidal behavior. In this sense, the results obtained in our context are consistent with a random-effects meta-analysis performed by Miranda-Mendizabal et al. [20], in which the importance of suicide prevention programs is evident. The importance of the gender variable is evidenced in these programs when discussing general and specific risk factors in young people. Specifically, this meta-analysis found a higher risk of attempted suicide in women and a higher risk of death by suicide in men, finding the following risk factors common to both men and women: mental disorder, previous substance abuse disorder, and previous exposure to interpersonal violence. To detect suicidal risk, knowing the coping strategies that a person would implement was fundamental, together with their emotional intelligence. A previous study demonstrated that the university environment was stressful for students, especially in their first years [89]. Hence, effective strategies for coping with emotions and the promotion of emotional education from childhood were required [90]. Another study revealed evidence of the relationships between the suicide risk variable and the stress coping strategies variable [48]. These types of evaluations were necessary to optimize the prevention of suicidal behavior [7] and address the issues from all angles [91]. It was also related to artificial intelligence and considered necessary before proceeding to the design of a program outside a university setting for preventive strategies. This may also contribute to better monitoring aspects and strategies, such as those recently commented on by Al-Halabí and Fonseca-Pedrero [22], wherein certain deficits, such as those related to emotional regulation, and a lack of psychological flexibility, such as cognitive flexibility, were relevant variables for vulnerability to suicidal behavior [22]. In addition, these types of evaluations and the book by Al-Halabí and Fonseca-Pedrero [22] can be useful to improve the strategy that responded to the acronym *PEPE* (*Ask*, *Listen*, *Promote Help and Be in Touch*) and the awareness and education proposed in the SOS program [92].

Other studies conducted on similar populations or Spanish-speaking populations include those by Castillo-Sánchez et al. [93], Matero et al. [79], and Vásquez-Escobar et al. [94]. In a recent review by Castillo-Sánchez et al. on the use of machine learning methods for suicide detection on social networks, Linguistic Inquiry and Word Count was found to be the most frequently used method for suicide risk prediction, according to the information posted on social networks, with machine learning-based models using the Python programming language being very useful in this process. The latter was implemented in 75% of the studies analyzed in this review, and the Supported Vector Machine was implemented in 65% of the studies, demonstrating the importance of the use of algorithms and models applied to social networks for suicide risk assessment. Similarly, another recent research [79] assessed suicidal risk using a dual context model (with suicide forum content and separated from other content) multilevel analysis of messages and users in forums. This study explored theoretical dimensions such as lexical emotional expression, personality characteristics, and the use of overt vocabulary, for example, in topics. To achieve this, among other aspects, they used a novel dual RNN (Recurrent Neural Network) architecture. They found that by combining suicide context and non-suicide elements, there were significant gains in building these predictive models. This combination allowed them to incorporate features of open vocabulary and theoretical dimensions, confirming the correlation between higher levels of anxious and depressive language with an increased risk for suicide, among other factors. Vásquez-Escobar et al.’s [94] cross-sectional analytical study on a Spanish-speaking population in Colombia examined factors associated with suicide attempts, specifically intentional intoxication; they found the highest risk associated with sociodemographic factors, with a higher risk for death in men than in women, as well as in people who were not part of a health insurance scheme. They also found that sociodemographic variables such as place of residence, sex, ethnicity, gender, age, and educational level were statistically significant when compared with mortality.

A limitation of this study was that self-reported questions were used. Hence, the responses may be subjective. The bias of having considered only the university population in a branch of knowledge, such as education, psychology, and pedagogy, could be a limitation of the study in terms of the generalization of the results. Similarly, the results were not compared with those of a no-university population. Given the relationship between educational level and suicide risk [95], the exclusive inclusion of university students may have influenced suicide-related factors. This may also have occurred due to the higher proportion of females included. However, determining the indicators of suicidal behavior and, thus, preventive measures is a social need for research, especially in the university setting, which according to some studies [90], can generate more stress and anxiety in young people. On a different note, a neural network requires an adequate amount of data to learn relevant patterns and features in the training data and to be able to generalize them correctly to new instances. Since the number of participants is limited, there could be problems in the generalization of the data, as well as an increased risk of overfitting the model to the training data and a bias in the conclusions [96]. It is also important to mention that dichotomization also has its disadvantages. For example, it can lead to a loss of information if the original variable is continuous in nature or has multiple categories with relevant information.

## 5. Conclusions

To conclude, the importance of this study was that it provided evidence with a methodology based on artificial intelligence that emotional care was a predictive variable in the ANN. Hence, interventions aimed at coping focused on emotional regulation and promotion of resilience in that evolutionary stage of emerging adulthood are required. This information, with the design of an artificial neural network with a predictive capacity of suicidal risk, is considered extremely useful and necessary in contrast with previous studies on this topic. Furthermore, this study confirmed the results using this methodology, which has many applications and advantages in clinical and educational psychology [52,97]. Additionally, using an artificial neural network instead of multivariate regression offers several advantages depending on the type of data and the research problem. Neural networks can learn complex nonlinear relationships between variables, thus overcoming the limitation of multivariate regression, which assumes linear relationships. In addition, neural networks can model abstract and hierarchical features automatically, whereas multivariate regression models require explicitly specifying features. They are highly adaptable and flexible to various types of data, such as images and text, and can handle large amounts of information more efficiently. Hidden layers in neural networks can learn relevant features without requiring manual selection, which is useful when domain knowledge is limited. In addition, they can generalize to complex problems such as image classification or natural language processing, where relationships are nonlinear. However, the higher computational requirement, the need for larger data for training, and the complexity in interpreting results compared with multivariate regression must be considered. The choice between the two methods will depend on the context and objectives of the research [96].

## Figures and Tables

**Table 1:** Descriptive statistics of the non-sociodemographic variables.

	*n*		Mean	Standard Deviation	Min.	Max.
	Valid	Lost				
Stress	159	178	21.42	10.879	0	42
Depression	160	177	15.98	11.468	0	42
Suicide risk ^a^	337	0	0.38	0.485	0	1
Perceived stress	232	105	29.87	8.616	5	54
Self-efficacy	163	174	28.25	4.880	10	40
Emotional care	256	81	27.56	6.528	10	40
Emotional clarity	256	81	27.35	6.312	8	40
Emotional repair	256	81	27.23	6.473	10	40
Anxiety state	264	73	25.18	12.378	0	59
Trait anxiety	265	72	28.89	7.945	5	54
Troubleshooting	264	73	12.47	5.056	0	20
Self-criticism	264	73	8.52	5.974	0	20
Emotional expression	264	73	11.75	5.190	0	20
Desiderative thinking	264	73	14.00	4.993	0	20
Social support	264	73	12.87	5.162	0	20
Cognitive restructuring	264	73	11.23	5.254	0	20
Problem avoidance	264	73	8.40	4.620	0	20
Social withdrawal	264	73	8.17	4.790	0	20
Perfectionism	163	174	99.62	24.605	48	166
High Sensitivity	163	174	124.08	25.59355	38.00	189.00

Note. ^a^: Dependent variable of the subsequent artificial neural network. Min: Minimum; Max: Maximum. Source: Own elaboration.

**Table 2:** Pearson correlations and confidence intervals of significant associations with the variable “Suicidal risk”.

	Pearson Correlation ^a^	Sig. (Bilateral)	95% Confidence Intervals (Bilateral) ^b^	
			Inferior	Superior
Number of siblings	0.020	0.843	−0.177	0.215
Average grade last year	0.032	0.750	−0.163	0.224
Emotional repair	0.035	0.642	−0.113	0.181
Anxiety state	−0.027	0.697	−0.164	0.110
Troubleshooting	0.011	0.877	−0.127	0.148
Emotional expression	0.039	0.578	−0.099	0.176
Desiderative thinking	−0.024	0.731	−0.161	0.114
Social support	0.048	0.497	−0.090	0.184
Cognitive restructuring	0.013	0.853	−0.125	0.150
Problem avoidance	0.046	0.515	−0.092	0.182
Social withdrawal	0.030	0.665	−0.107	0.167

Note. ^a^: Significance value *p* < 0.05; ^b^: Estimation is based on Fisher’s r to z transformation with bias adjustment. Source: Own elaboration.

**Table 3:** Group statistics as a function of the presence or absence of suicidal risk.

	Suicide Risk	*n*	Mean	Standard Deviation
Stress	No	72	21.86	11.215
	Yes	87	21.06	10.644
Depression	No	72	16.61	11.951
	Yes	88	15.45	11.099
Anxiety	No	69	17.45	12.347
	Yes	87	15.61	11.415
Suicide risk	No	92	2.85	1.482
	Yes	127	10.24	3.014
Perceived stress	No	114	28.90	8.660
	Yes	118	30.81	8.507
Family loneliness	No	67	19.75	4.110
	Yes	90	19.82	3.740
Romantic solitude	No	67	18.67	4.467
	Yes	90	19.30	4.035
Self-efficacy	No	73	28.93	4.617
	Yes	90	27.69	5.041
Emotional care	No	135	25.57	5.701
	Yes	121	29.78	6.698
Emotional clarity	No	135	27.28	5.591
	Yes	121	27.43	7.053
Emotional repair	No	135	27.46	6.521
	Yes	121	26.98	6.438
Anxiety state	No	140	23.79	12.849
	Yes	124	26.75	11.678
Trait anxiety	No	141	26.68	8.698
	Yes	124	31.41	6.113
Troubleshooting	No	141	12.86	5.065
	Yes	123	12.02	5.030
Self-criticism	No	141	7.65	6.184
	Yes	123	9.53	5.580
Emotional expression	No	141	11.74	4.929
	Yes	123	11.77	5.493
Desiderative thinking	No	141	14.04	5.020
	Yes	123	13.94	4.982
Social support	No	141	13.27	4.799
	Yes	123	12.41	5.534
Cognitive restructuring	No	141	11.65	5.114
	Yes	123	10.75	5.391
Problem avoidance	No	141	8.56	4.799
	Yes	123	8.21	4.417
Social withdrawal	No	141	8.02	4.789
	Yes	123	8.34	4.806
Perfectionism	No	73	103.97	27.251
	Yes	90	96.09	21.751
High Sensitivity	No	73	130.6986	25.15214
	Yes	90	118.7222	24.81030

Source: Own elaboration.

**Table 4:** Independent samples test for suicide risk.

Cohen’s *d*		*t*	*df*	*p*	Difference in Averages	Standard Error Difference	95% CI	
							Inferior	Superior
Perceived stress	−0.222	−1.687	230	0.046	−1.902	1.127	−4.122	0.319
Romantic solitude	−0.149	2.044	157	0.021	1.584	0.775	0.053	3.115
Emotional care	−0.679	−5.379	236.967	<0.001	−4.206	0.782	−5.747	−2.666
Anxiety state	−0.240	−1.948	262	0.026	−2.957	1.518	−5.947	0.033
Trait anxiety	−0.623	−5.168	251.225	<0.001	−4.730	0.915	−6.533	−2.928
Self-criticism	−0.319	−2.582	262	0.005	−1.883	0.729	−3.319	−0.447
Perfectionism	0.324	2.054	161	0.021	7.884	3.838	0.305	15.462
High Sensitivity	0.480	3.046	161	0.001	11.97641	3.93207	4.21133	19.74149

Source: Own elaboration.

**Table 5:** ANN parameter estimates.

Predictor		Forecast		
		Hidden Layer 1	Output Layer	
		H(1:1)	[SR = 0]	[SR = 1]
Input layer	(Bias)	−0.866		
	[Gender = 1]	−0.596		
	[Gender = 2]	−0.289		
	Age	0.215		
	Depression	−0.142		
	Stress	−0.533		
	Self-efficacy	0.454		
	Care	−1.641		
	Clarity	−0.408		
	Repair	0.112		
	State	0.539		
	Trait	−0.899		
	PS	−0.171		
	SC	0.350		
	EE	−0.047		
	DT	0.306		
	SS	−0.081		
	CR	0.322		
	PA	0.744		
	SW	1.249		
	P	1.125		
	HS	−0.125		
Hidden layer 1	(Bias)		−0.801	0.736
	H(1:1)		1.591	−2.131

Note: Sex = 1: Male; Sex = 2: Female; Care: Emotional care; Clarity: Emotional clarity; Repair: Emotional repair; State: Anxiety state; Trait: Anxiety trait; PS: Problem solving; SC: Self-criticism; EE: Emotional expression; DT: Desiderative thinking; SS: Social support; CR: Cognitive restructuring; PA: Problem avoidance; SW: Social withdrawal; P: Perfectionism; HS: High Sensitivity. SR = 0: absence of suicidal risk; SR = 1: presence of suicidal risk. Source: Own elaboration.

**Table 6:** Significance of the ANN’s independent variables.

	Importance	Standardized Importance
Emotional care	0.183	100.0%
Social withdrawal	0.134	73.5%
Perfectionism	0.117	64.1%
Trait anxiety	0.082	45.1%
Problem avoidance	0.075	41.2%
Anxiety state	0.055	30.0%
Total perceived stress	0.054	29.5%
Self-efficacy	0.043	23.5%
Emotional clarity	0.041	22.7%
Self-criticism	0.035	19.3%
Cognitive restructuring	0.031	17.2%
Sex	0.030	16.2%
Desiderative thinking	0.029	16.0%
Age	0.022	11.8%
Troubleshooting	0.017	9.3%
Depression	0.014	7.5%
High Sensitivity	0.012	6.8%
Emotional repair	0.011	6.0%
Social support	0.008	4.4%
Emotional expression	0.005	2.5%

Source: Own elaboration.

**Table 7:** Classification of cases by phase.

Phase	Observed	Forecast		
		No	Yes	Percent Correct
Training	No	4	4	50.0%
	Yes	5	49	90.7%
	Overall percentage	14.5%	85.5%	85.5%
Tests	No	0	1	0.0%
	Yes	0	21	100.0%
	Overall percentage	0.0%	100.0%	95.5%
Reservation	No	1	1	50.0%
	Yes	1	11	91.7%
	Overall percentage	14.3%	85.7%	85.7%

Note: Dependent variable: dichotomous suicidal risk. Missing data are excluded. Source: Own elaboration.

## Data Availability

The data can be requested by the scientific community in the ethical terms to be determined.
